# Dermoscopy and Ultraviolet-Enhanced Fluorescence Dermoscopy (UEFD) Increase the Accuracy of Diagnosis and Are Useful in Assessing the Effectiveness of *Kerion celsi* Treatment

**DOI:** 10.3390/jof11010052

**Published:** 2025-01-09

**Authors:** Justyna Putek, Danuta Nowicka, Alina Jankowska-Konsur

**Affiliations:** 1 University Centre of General Dermatology and Oncodermatology, Wroclaw Medical University, 50-368 Wroclaw, Poland; 2 Division of Aesthetic Dermatology and Regenerative Medicine of the Skin, Wroclaw Medical University, 50-368 Wroclaw, Poland

**Keywords:** fungal infection, *Kerion celsi*, alopecia, *Microsporum canis*, dermatophyte, zoonosis

## Abstract

*Microsporum canis*, a zoophilic dermatophyte, infects the stratum corneum and keratinized tissues like hair and nails in cats and dogs, with cats serving as the primary reservoir. Most human infections arise from animal contact. We present the case of a girl aged 8 with skin scalp lesions persisting for two months. Several scalp lesions, with a maximum diameter of 4 cm, presented as erythematous plaques with superficial scaling, yellow crusts, and edematous areas with purulent exudate. Dermoscopy revealed yellow crusts on an erythematous background, along with white scales, pustules, broken hairs, and comma hairs. Ultraviolent-enhanced fluorescence dermoscopy (UEFD) showed slight celadon green fluorescence, which enhanced the diagnosis and further helped to monitor the treatment. The PCR test confirmed the presence of *M. canis*. Treatment included topical ciclopirox and oral terbinafine. Lesions on the scalp and noticeable hair regrowth were observed in the areas of hair loss after two months. *Kerion celsi* can result in severe alopecia. To prevent scarring associated with hair loss in children, early mycological diagnostics, supported by dermoscopy and UEFD, is recommended.

## 1. Introduction

Dermatophyte infections are commonly distributed worldwide. *Microsporum canis* is a fungus that is generally considered a zoophilic dermatophyte, and it infects the stratum corneum of the epidermis and keratinized tissues such as nails and hair of domesticated cats and dogs. *M. canis* is the most frequent pathogen causing tinea capitis in children in Europe, with the highest incidence of infections during hot months. Cats are regarded as the most important reservoir hosts. Most human infections are acquired from animals [1].

Tinea capitis is an infection of the scalp caused by dermatophytes such as *Microsporum* and *Trichophyton* species [1], and it occurs mainly in prepubertal children [2]. Adults can occasionally be affected [3]. Clinically, tinea capitis presents scaly patches with a well-defined area of hair loss, the additional presence of black dots at the follicular opening, and diffuse scalp scaling with subtle hair loss [4]. The severe form of tinea capitis is called *Kerion celsi* and is caused mainly by *Trichophyton tonsurans*, *Trichophyton verrucosum*, and *Trichophyton mentagrophytes*, and occasionally by *M. canis* [5,6]. *Kerion celsi* is a severe inflammatory reaction with painful, erythematous, boggy plaques, which leads to the destruction of hair follicles and often to scarring alopecia; therefore, early diagnosis of this condition is important. Timely diagnosis is essential to prevent complications. In this context, dermoscopy and its advanced features can be valuable tools for differential diagnosis, guiding therapeutic decisions, and monitoring treatment effectiveness. Dermoscopy is particularly useful for rapid initial assessments, especially in situations where mycological tests are unavailable [7]. Recent technological advancements have introduced a novel modification to this technique by incorporating ultraviolet (UV) light. This innovation, known as ultraviolet-enhanced fluorescence dermoscopy (UEFD), integrates UV light into dermatoscopes, enhancing the differential diagnosis of various skin conditions, including infections, alopecia, vitiligo, melasma, porokeratosis, psoriasis, and neoplastic processes. Certain pathogens produce fluorochromes—fluorescent chemical molecules capable of re-emitting light upon exposure to ultraviolet (UV) light [8]. This property allows for their visualization and differentiation in biological samples. Studies have shown that the color of ultraviolet-induced fluorescence is specific to particular species of infection-causing pathogens [9]. For instance, *M. canis*, the causative agent of tinea corporis, produces pteridine, which fluoresces yellow to green. In contrast, *Malassezia furfur*, which is responsible for pityriasis versicolor, produces pityrialactone, which fluoresces light green [8]. We present a case report of a girl who developed *Kerion celsi* caused by *M. canis*.

## 2. Case Description

**Figure 1:**
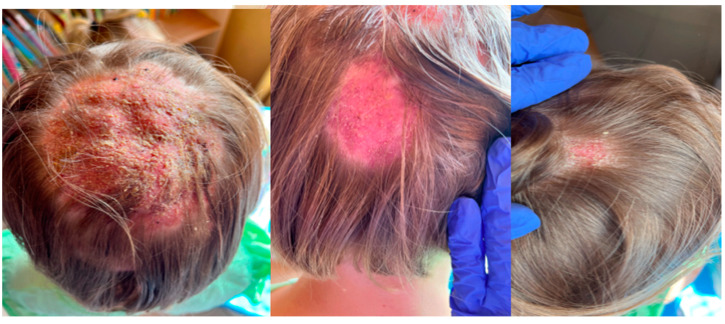
The clinical presentation of *Kerion celsi* in an 8-year-old girl on the day of admission (left lesion: 4 cm × 4 cm, central scalp; middle: 1.5 cm × 1 cm, parietal scalp; right: 0.5 cm × 0.5 cm, parietal scalp).

**Figure 2:**
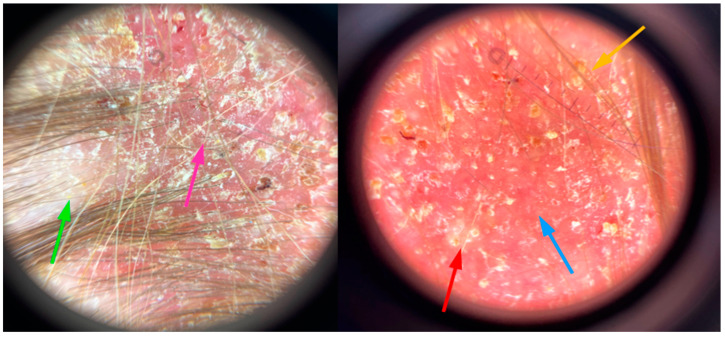
The dermoscopic presentation of *Kerion celsi* in an 8-year-old girl on the day of admission. Green arrow—white scales, pink arrow—broken hairs, red arrow—pustules, blue arrow—erythema, orange arrow—yellow crusts.

**Figure 3:**
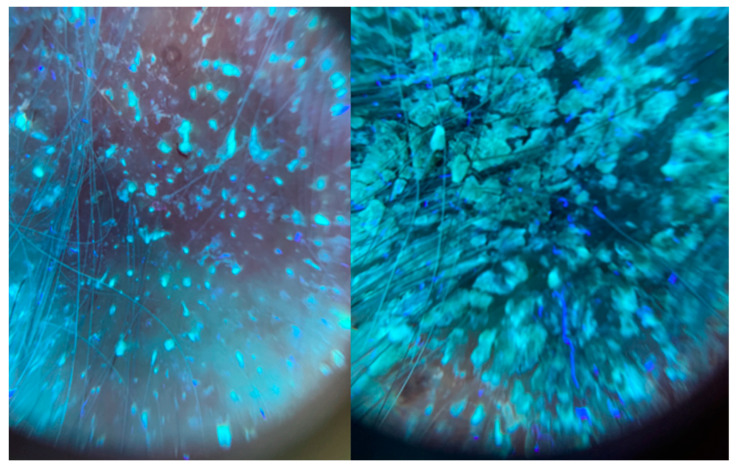
The ultraviolet-enhanced fluorescence dermoscopy of *Kerion celsi* in an 8-year-old girl on the day of admission. Celadon green perifollicular fluorescence can be observed.

The girl, aged 8, presented to the Department of Dermatology with scalp lesions persisting for two months. The patient presented with three areas of hair loss in the parietal and occipital parts of the scalp. First, the most severe lesion was localized in the central part of the scalp and measured 4 cm × 4 cm (Figure 1). The lesion was described as erythematous plaques with superficial scaling, yellow crusts, and edematous regions with purulent exudate. The patient reported mild tenderness on palpation. The second lesion, located on the parietal region of the scalp, was oval in shape and measured 1.5 cm × 1 cm. It was described as an erythematous plaque with scaling and yellow-to-red crusts. The last erythematous plaque lesion was the smallest, round, with a diameter of 0.5 cm and localized on the parietal part of the scalp. The two smaller lesions caused mild pruritus. Following dermoscopy, yellow crusts on an erythematous background, white scales, pustules, broken hairs, and comma hairs were found (Figure 2). One of the simple and painless tools we used to expedite and facilitate diagnosis was Wood’s lamp, a device emitting long-wave ultraviolet light (365 nm) to detect the fluorescence of certain dermatophytes. Modern dermatoscopes, such as the Dermlite DL5, incorporate a built-in Wood’s lamp, enhancing the diagnosis of fungal infections. Using UEFD (peak wavelength 365 nm produced by Dermlite LLC, Aliso Viejo, CA, USA), we observed slight celadon green perifollicular fluorescence (Figure 3). Additionally, samples were taken for a mycological examination and PCR testing before starting treatment, as part of the diagnostic process.

**Figure 4:**
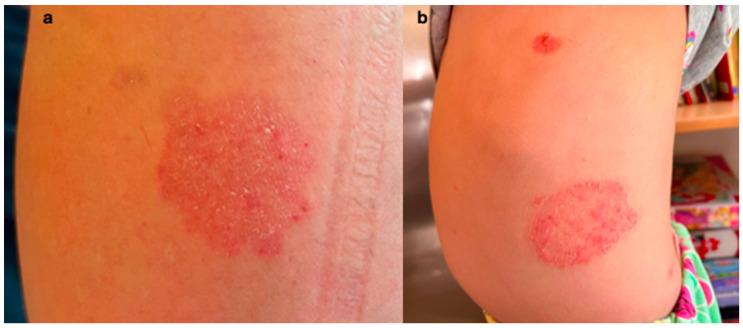
The clinical presentation of tinea corporis in the mother and the younger sister of the patient: (**a**) skin lesion on the left lower limb of the mother, and (**b**) skin lesions on the trunk of the younger sister.

Information about the skin lesions experienced by family members added valuable context to this case and could help in differential diagnosis. The mother of the patient reported that the younger sister and brother of the girl also had erythematous, oval plaques, and sharply demarcated lesions with a raised edge on the trunk and on the back, which appeared around two months before (Figure 4). Furthermore, similar lesions also appeared on the skin of the lower limbs of the patient’s mother (Figure 4). The siblings and the mother, before admission to our clinic, were treated by a family doctor with topical ciclopirox and the 8-year-old girl was referred to our department.

After taking a medical history, it turned out that the parents purchased a guinea pig three months before the lesions appeared and the children played intensively with it. The patient admitted that she put the guinea pig on her head while playing. It is worth noting that the younger sister and brother, who denied putting a supposedly infected guinea pig on their heads, did not develop any symptoms of tinea capitis.

Furthermore, the mother reported that recently, the children petted some homeless cats. It was impossible to test the animals for fungal infections because the guinea pig was returned to the pet shop after skin changes appeared in the children and the cats were unapproachable. We raised the suspicion of *Kerion celsi* in the 8-year-old girl and tinea corporis in the siblings and the mother, most probably caused by the same dermatophyte.

**Figure 5:**
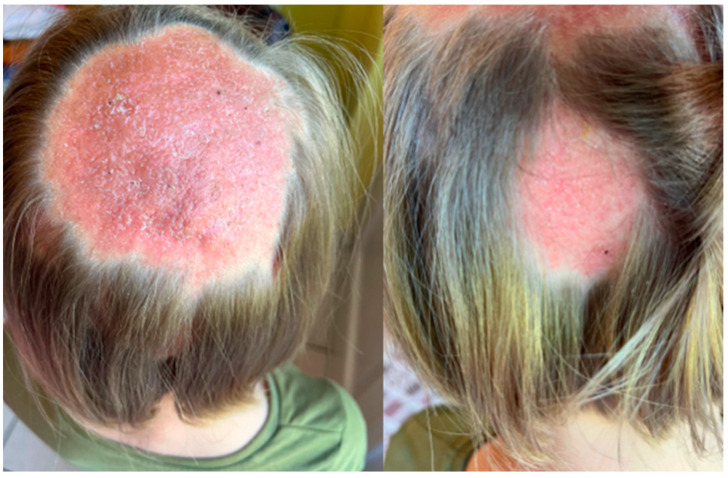
The clinical presentation of *Kerion celsi* in an 8-year-old girl after one month of follow-up.

**Figure 6:**
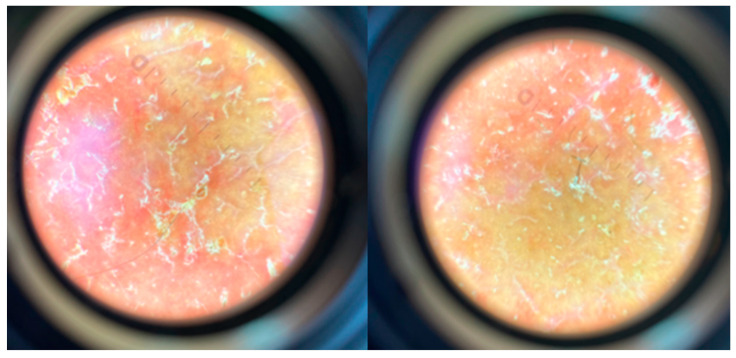
The dermoscopy findings of *Kerion celsi* in an 8-year-old girl after one month of follow-up.

**Figure 7:**
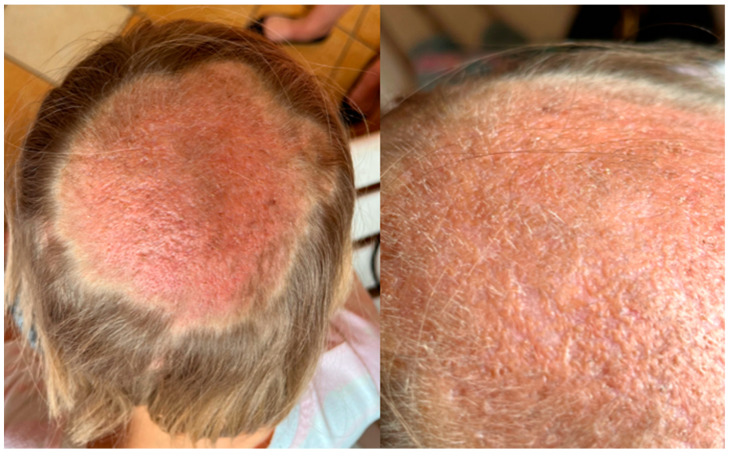
The dermoscopy findings of *Kerion celsi* in an 8-year-old girl after two months of follow-up.

**Figure 8:**
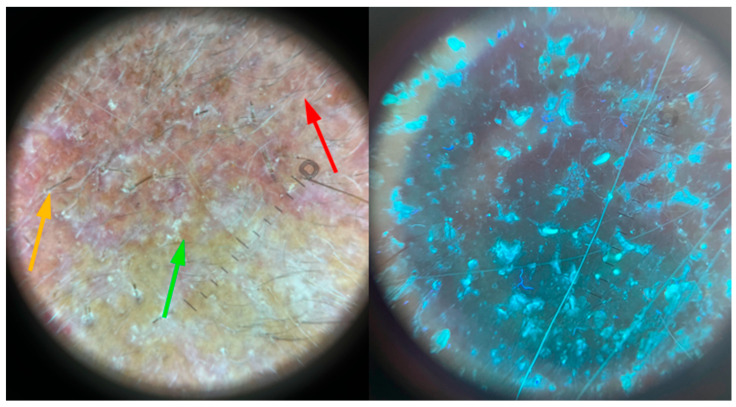
The dermoscopy and ultraviolet-enhanced fluorescence dermoscopy findings of *Kerion celsi* in an 8-year-old girl after two months of follow-up. Green arrow—white scales with post-inflammatory hyperpigmentation, orange arrow—broken hairs, red arrow—vellus hair.

**Figure 9:**
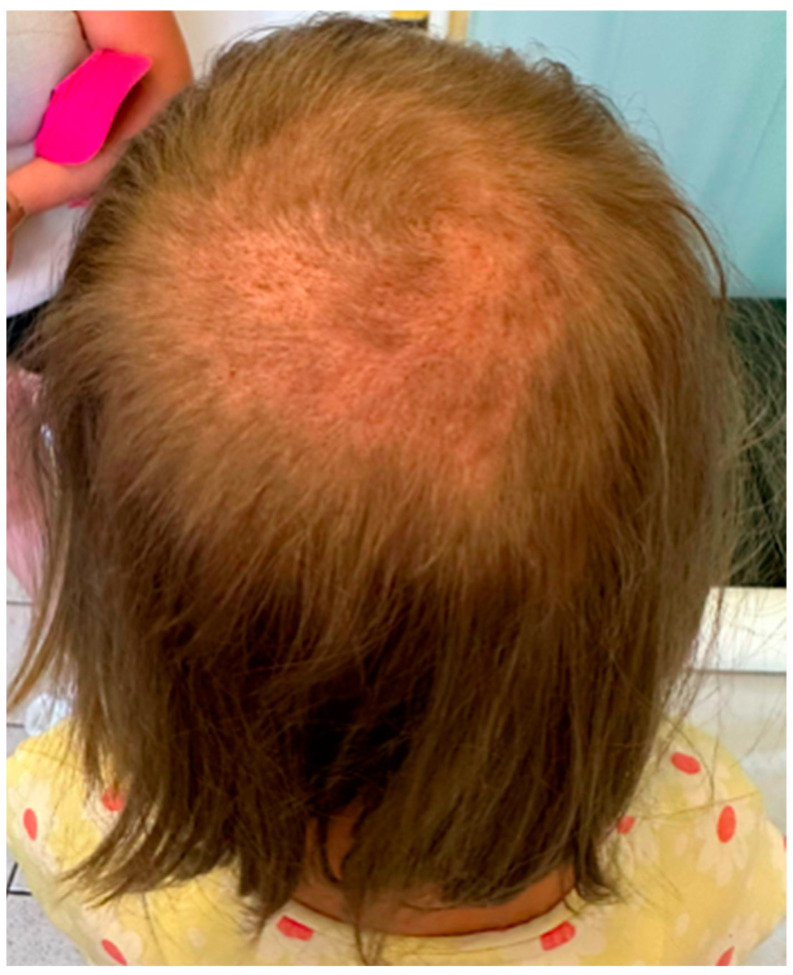
The clinical presentation of *Kerion celsi* in an 8-year-old girl after a three-month follow-up period.

The PCR test and mycological examination were positive for *M. canis*. Treatment was started and the patient was treated with a combination of topical ciclopirox and oral terbinafine (125 mg, once daily) for 12 weeks. The girl came back for follow-up one month later and there was already a visible improvement. In the lesions on the scalp of the patient, the scales and crusts had been reduced and the formed abscesses had become less palpable (Figure 5). In the dermoscopy results, no hair regrowth was observed (Figure 6). We decided to continue the antifungal treatment. After 4 weeks, the girl was admitted again for the follow-up. The lesion on the center of the scalp of the patient had become less inflammatory, and both clinically (Figure 7) and dermoscopically (Figure 8), hair regrowth was noticed. Still, slight celadon green fluorescence was observed. The treatment was continued. The girl presented again for follow-up after one month (Figure 9). The hair regrowth was more noticeable, and in the UEFD results, no celadon green fluorescence was observed. The treatment was completed after 12 weeks. At this timepoint, the mycological examination was negative.

## 3. Discussion

Tinea capitis can have a polymorphic clinical presentation, which can be easily confused with bacterial folliculitis, folliculitis decalvans, or dissecting cellulitis [10]. This can lead to delayed diagnosis and delayed treatment, which can result in scarring associated with hair loss [7]. The diagnosis of tinea corporis and tinea capitis is usually made based on medical history and physical examination; however, to confirm the diagnosis, a mycological examination is always required [11]. The important clues that raise suspicion of tinea are contact with rodents, such as guinea pigs, hamsters, and degus, and other animals, such as mammals (cats and dogs). It is worth noting that pet guinea pigs are often asymptomatic carriers of contagious pathogens, making this source of infection easy to overlook [12]. In the differential diagnosis, the conditions to consider include bacterial folliculitis, seborrheic dermatitis, bacterial cellulitis, cutaneous tuberculosis, scalp psoriasis, alopecia areata, and scarring associated with lupus erythematosus.

The mycological examination, which implies direct microscopic examination and culture, can confirm the diagnosis of tinea; nonetheless, access to these kinds of tests is limited in some countries [11]. Additionally, the mycological cultures take up to 4 weeks or longer [1], which delays proper diagnosis. The periodic acid–Schiff (PAS) staining method is a valuable diagnostic tool for *Kerion celsi*, as it enables the visualization of fungal elements within tissue samples, aiding in the accurate identification of the causative organism.

Of note, several novel molecular tools were developed for the rapid identification of dermatophytes [13,14]. For example, a commercial multiplex real-time PCR test enables clinicians to diagnose fungal infection within 48 h [14,15]. The test is highly specific; however, the costs are high, which results in limited accessibility. PCR is less accessible than microscopic examination.

The other techniques that are useful in diagnosing fungal infections are in vivo reflectance confocal microscopy, which is rarely used in daily practice due to its high cost, and skin biopsy. Skin biopsy is performed mainly when diagnostic doubts occur and to differentiate from other dermatoses, such as psoriasis or seborrheic dermatitis [16]. It is worth noting that this technique is invasive and that the provided histopathological picture is often unspecific [11].

The helpful tools, apart from the mycological test and the above-described techniques, are dermoscopy and UEFD, which are performed to enhance the diagnosis and to monitor the treatment. Most characteristic trichoscopic findings of tinea capitis are non-specific: broken hairs, black dots, and perifollicular or interfollicular scaling, but more specifically: comma hairs, corkscrew hairs, Morse-code-like hairs, zigzag hairs, and bent hairs [17].

There are some differences in the dermoscopy of *Microsporum* spp., which typically grow within the hair follicle and cover the surface of the hair shaft (ectothrix invasion), compared to *Trichophyton* spp. which generally grow within the hair shaft only (endothrix invasion) [1].

According to Waśkiel-Burnat et al. [18], Morse-code-like hairs, zigzag hairs, bent hairs, and diffuse scaling are present mainly in tinea capitis caused by *Microsporum* spp., and conversely, corkscrew hairs are more commonly observed in tinea capitis caused by *Trichophyton* spp.

The dermoscopy of *Kerion celsi* is slightly different because of its more inflammatory course. In the dermoscopy results, erythema, broken hairs, black dots, perifollicular scaling, pustules, and yellow crusts can be observed [7,18,19].

In the UV-dermoscopy results, *M. canis* can exhibit yellow to green fluorescence due to fluorochrome pteridine [8]. The UV-dermoscopy of the skin lesions caused by *Trichophyton* spp. show no fluorescence [20]. UEFD, according to Errichetti et al. [20], seems to be more accurate in differentiating dermatophyte infections and can be used in relation to atypical forms of many dermatological conditions, which can add diagnostic confidence while examining the patient [8]. Further, Errichetti et al. [20] reported that green, follicular fluorescence is highly specific for *Microsporum* spp. infections compared to *Trichophyton* spp. infections.

In our case, celadon green follicular fluorescence was observed on the day of admission and declined with the course of time and treatment, and finally, after 12 weeks, it disappeared. This fact helped us to decide to stop the treatment.

Tinea capitis is often treated with oral antifungal agents such as griseofulvin, terbinafine, and itraconazole. A course of a minimum of 6–8 weeks is often advised. According to Gupta et al. [1], griseofulvin more effectively treats *Microsporum* spp. infections, while terbinafine and itraconazole more effectively cure *Trichophyton* infections. Chiriac et al. [21] used griseofulvin in an 11-year-old boy with *Kerion celsi* caused by *M. canis*, which resulted in complete mycological remission after 8 weeks. Aste et al. [22] used terbinafine in a newborn with *Kerion celsi* caused by *M. canis* with success. In our case, we used terbinafine due to the limited access to griseofulvin in our country and the lack of registration of itraconazole for children.

Treatment with terbinafine requires special attention due to its lower effectiveness compared to griseofulvin. Furthermore, the treatment course when using terbinafine is often longer. Our patient was treated successfully with terbinafine, with complete recovery, which was confirmed by the lack of fluorescence in the UEFD results; however, at the last follow-up, the patient found the hair regrowth unsatisfactory, as full hair restoration had not been achieved.

The prognosis of hair regrowth after *Kerion celsi* is rather unknown and not well documented. In the papers by Bonven et al. [23] and Foged et al. [24], respectively, only five and four out of eighteen patients had normal hair growth after *Kerion celsi*.

## 4. Conclusions

*Kerion celsi* is a severe inflammatory form of tinea capitis, where delayed diagnosis and treatment can lead to serious consequences, such as scarring associated with hair loss. Although dermoscopy and UEFD cannot replace standard mycological examination in diagnosing and identifying fungal pathogens, they are rapid, cost-effective, and efficient auxiliary diagnostic methods that assist in therapeutic decisions and patient follow-up.

## Data Availability

Data are contained within the article.
